# A Simple Method for the Fabrication of Silicon Inverted Pyramid Substrates for Surface-Enhanced Raman Spectroscopy

**DOI:** 10.3390/ma16103634

**Published:** 2023-05-10

**Authors:** Jia Liu, Yao Yan, Zimu Zhang, Yuchen Liu, Jia Ge, Zisheng Guan

**Affiliations:** College of Materials Science and Engineering, Nanjing Tech University, 30 South PuZhu Road, Nanjing 210009, China; 202061203272@njtech.edu.cn (J.L.); 202061203187@njtech.edu.cn (Y.Y.); 202161203162@njtech.edu.cn (Z.Z.); 201861103001@njtech.edu.cn (Y.L.); ge.jia@njtech.edu.cn (J.G.)

**Keywords:** silicon Figure Sinverted pyramids, silver-assisted chemical etching, silver nanoparticles, surface-enhanced Raman spectroscopy

## Abstract

Silicon inverted pyramids have been shown to exhibit superior SERS properties compared to ortho-pyramids, yet low-cost, simple preparation processes are lacking at present. This study demonstrates a simple method, silver-assisted chemical etching combined with PVP, to construct silicon inverted pyramids with a uniform size distribution. Two types of Si substrates for surface-enhanced Raman spectroscopy (SERS) were prepared via silver nanoparticles deposited on the silicon inverted pyramids by electroless deposition and radiofrequency sputtering, respectively. The experiments were conducted using rhodamine 6G (R_6_G), methylene blue (MB) and amoxicillin (AMX) molecules to test the SERS properties of the Si substrates with inverted pyramids. The results indicate that the SERS substrates show high sensitivity to detect the above molecules. In particular, the sensitivity and reproducibility of the SERS substrates with a denser silver nanoparticle distribution, prepared by radiofrequency sputtering, are significantly higher than those of the electroless deposited substrates to detect R_6_G molecules. This study sheds light on a potential low-cost and stable method for preparing silicon inverted pyramids, which is expected to replace the costly commercial Klarite SERS substrates.

## 1. Introduction

Surface-enhanced Raman scattering spectroscopy (SERS) is a spectroscopic analysis technique with great technical advantages, such as ultra-high sensitivity, fingerprinting features, a fast response, and a non-destructive nature [1,2,3,4]. It has been widely applied in various fields across different industries, e.g., food safety, bioanalysis, and drug testing [5,6,7]. Researchers believe that the SERS enhancement mechanism can be divided into electromagnetic enhancement (EM) and chemical enhancement (CM), and claim that EM enhancement plays a dominant role. EM enhancement is caused by localized surface plasmon resonance (LSPR) generated between metal nanostructures or nanoparticles [8,9]. For applications of SERS, highly reproducible, large-scale production and low-cost substrates remain a challenge [10,11]. The study of SERS substrates has been expanded from the initial precious metal materials to semiconductor materials, such as Si, due to its excellent optoelectronic properties and stability. However, the enhanced Raman effect of pure silicon is not as sensitive as that of precious metals; therefore, the synergistic effect of precious metals is needed [12,13,14]. It has previously been demonstrated that 3D structured silicon with a large specific surface area and controlled 3D structure can effectively increase the number of hot spots and further improve the sensitivity of SERS signals [15,16].

A large number of researchers have so far prepared SERS substrates in different 3D structures of silicon, such as nanowires [17,18], nanopores [19], nanocones [20], pyramids [21,22,23], and inverted pyramids [24,25]. Silicon inverted pyramids with anti-reflective properties favor multiple interactions of incident light compared to flat silicon and increase light absorption and the number of adsorption hot spots. Nevertheless, a limited number of studies on inverted pyramidal SERS substrates have been conducted, and only a few scholars have purchased commercial clathrate substrates to explore the SERS properties of inverted pyramids [26]. Furthermore, the conventional method of etching silicon inverted pyramids is often tedious, the preparation cost of plasma etching is considerably high, and the commercially available Klarite SRES substrates with inverted pyramid structures are quite expensive [11,27]. Thus, taking all these factors into consideration, it is of great interest to explore a low-cost and simple method to texturize silicon inverted pyramid SERS substrates to substitute expensive commercial inverted pyramid SERS substrates (Klarite).

Our previous study proposed a novel metal-assisted chemical etching system based on silver-assisted chemical etching combined with polyvinylpyrrolidone synergy (Ag@PVP-ACE) to produce inverted pyramidal silicon with anti-reflective properties. We demonstrated that this method greatly simplified the process and reduced the cost compared to the conventional etching of inverted pyramidal silicon [27,28,29,30]. In this paper, we finalized the experimental parameters that can be used to prepare silicon inverted pyramids with excellent anti-reflectivity and size uniformity. The SERS substrates were prepared by depositing silver nanoparticles (Ag NPs) onto the surface of the inverted pyramidal silicon using the electroless deposition method [20,31] and a radiofrequency sputtering system [1]. The SERS sensitivity at different silver deposition states was investigated with R_6_G [32]. In addition, MB and AMX molecules were used to verify the universal applicability of the SERS substrate [1,33]. The outcomes of this study may provide a useful guide for the construction of inverted pyramidal structured silicon SERS substrates with high reproducibility, high sensitivity, and a low cost.

## 2. Experimental Section

### 2.1. Materials

The experiments conducted in this study were based on diamond wire saw (DWS) (100) oriented p-type silicon wafers (156.75 × 156.75 mm^2^, thickness 180 ± 10 μm, resistivity 0.5–1.5 Ω·cm). We obtained silver nitrate (AgNO_3_, AR) from Sinopharm Chemical and purchased ammonia (NH_3_·H_2_O, 25%, AR) and hydrogen peroxide (H_2_O_2_, 30%, GR) from Shanghai Lingfeng Chemical Reagent Co., Shanghai, China. High-purity deionized water with resistivity of 18.25 MΩ·cm^−1^ was used during the experiments, and the necessary dose of ammonium hydrogen fluoride (NH_4_HF_2_, AR) was purchased from Nanjing Chemical Reagent Co., Nanjing, China. Polyvinylpyrrolidone (PVP-K88-96, AR), rhodamine 6G (R_6_G, AR), and methylene blue (MB, AR) were provided by Shanghai Aladdin Biochemical Technology Co., Shanghai, China. All chemicals were purchased and used directly.

### 2.2. Fabrication of Silicon Inverted Pyramid/Ag NPs

**Figure 1:**
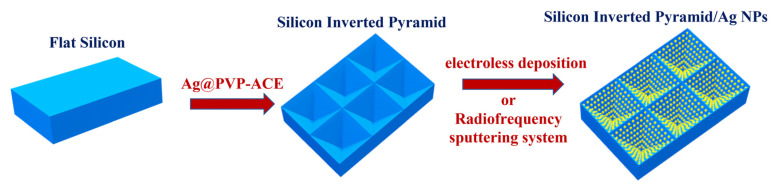
Schematic procedure for the fabrication of silicon inverted pyramid/Ag NPs.

Figure 1 illustrates the fabrication process of the silicon inverted pyramid/Ag NP SERS substrate. Silver-assisted chemical etching was used for the experiments, and the optimal reaction temperature was set at 65 °C, following the standards of our previous research [28]. Before etching, all silicon wafers had to be primarily polished to form flat silicon. Texturing of the flat silicon wafers with an etching solution of NH_4_HF_2_, H_2_O_2_, PVP, and AgNO_3_ was performed at 65 °C for a certain time. Then, the Ag NPs remaining on the silicon surface were removed with a solution of 5% NH_3_·H_2_O/5% H_2_O_2_, followed by a deionized rinse of the wafer for 1 min. Different experimental parameters were adjusted to explore their effects on the etched inverted pyramid morphology. The silicon inverted pyramid substrate with anti-reflective properties facilitates multiple interactions of the incident light, while the uniform structure of the substrate facilitates the homogeneity of the Raman data.

The silicon inverted pyramids were decorated with silver nanoparticles using the following methods: (1) electroless deposition by immersing the textured Si wafers in AgNO_3_ (0.5 mM) and HF (9% *v*/*v*) aqueous solution for different time periods, and then washing the wafers with deionized water, followed by air drying at room temperature; (2) in a radiofrequency sputtering system, the MS600B high-throughput multi-target magnetron sputter coater with a sputtering power of 15 w was selected to deposit silver nanoparticles on the textured Si wafers.

### 2.3. Characterization

The surface micromorphology of the Si wafers was observed by scanning electron microscopy (SEM, JSM-IT200, Tokyo, Japan) and field emission scanning electron microscopy (FESEM, Hitachi, 4800, Tokyo, Japan) at an accelerating voltage of 5 kV. After this, it was possible to measure the reflectance spectra of the samples in the wavelength range of 350–1050 nm with a UV–Vis–NIR spectrophotometer (UV-3101PC, Tokyo, Japan).

### 2.4. SERS Measurements

Rhodamine 6G (R_6_G) molecules were used as chemical targets to evaluate the SERS responses of the silicon inverted pyramid/Ag NPs substrates. In addition, we used methylene blue (MB) and amoxicillin (AMX) molecules to demonstrate the universal applicability of this substrate. R_6_G, MB, and AMX molecules were diluted in deionized water. The silicon inverted pyramid/Ag NP substrates were immersed in different concentrations of target solutions at room temperature for 30 min in the dark, and then dried naturally at room temperature. The Raman microscope system (HORIBA XploRA PLUS, Shanghai, China) was equipped with a 532 nm laser to acquire SERS spectra. For Raman measurements, the test conditions were consistent for all samples, with an acquisition time of 30 s, an acquisition exposure time of 1 s, an irradiation power of 1 mW for the laser, and a 50× objective with a 0.5 NA (numerical aperture) value. SERS spectra were collected from different regions of the samples to ensure data accuracy. Furthermore, bare silicon with a flat surface and no metal nanoparticles was used as a reference sample for normal Raman spectroscopy with the R_6_G concentration of 0.1 M.

## 3. Results and Discussion

### 3.1. Evaluation of Silicon Inverted Pyramid

**Figure 2:**
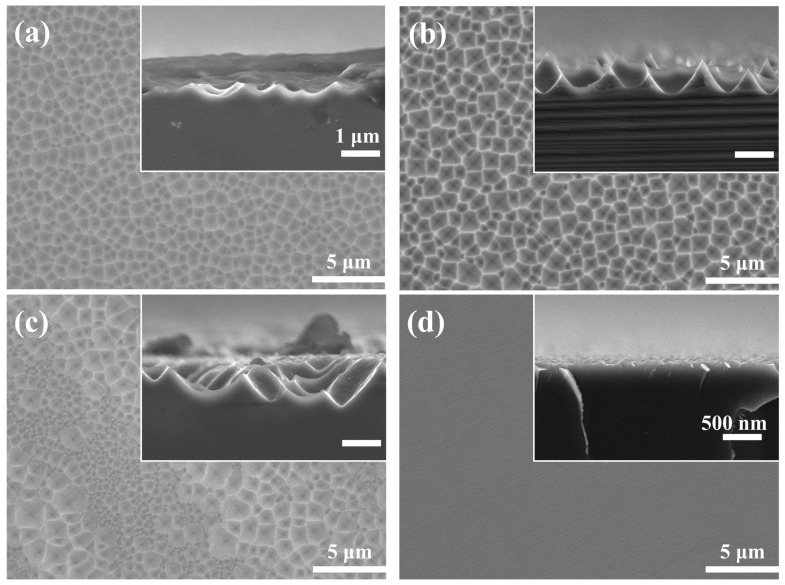
SEM images of Si surface and cross-section etched in different NH_4_HF_2_ solutions for 7 min: (**a**) 0.3 M; (**b**) 0.5 M; (**c**) 0.7 M; (**d**) 0.9 M.

Figure 2 shows the surface morphology of the silicon wafer etched in different NH_4_HF_2_ solutions for 7 min. When the NH_4_HF_2_ concentration was 0.3 M, the Si surface was covered by a shallow crater structure and the sidewall edges were blurred (Figure 2a). With increasing NH_4_HF_2_ concentrations (0.5 M), the shallow crater structure on the silicon surface transformed into a large-scale, randomly distributed, inverted pyramidal structure with a size and depth of approximately 1 μm (Figure 2b). Further increasing the NH_4_HF_2_ concentration led to polished surfaces on the silicon wafer surface; the etched structure was no longer uniform, a large number of small-sized inverted pyramids were interspersed between the large-sized inverted pyramid structures, and the side edges became blurred (Figure 2c). When the concentration reached 0.9 M, one face of the wafer was completely converted into a polished surface with a flatter surface, as illustrated in Figure 2d. The reflectance of silicon wafers etched with different concentrations of NH_4_HF_2_ is shown in Figure S1. When the NH_4_HF_2_ concentration was 0.5 M, the reflectivity of the silicon wafer was the lowest. Hence, we concluded that the concentration of NH_4_HF_2_ in the etching solution system should be 0.5 M.

**Figure 3:**
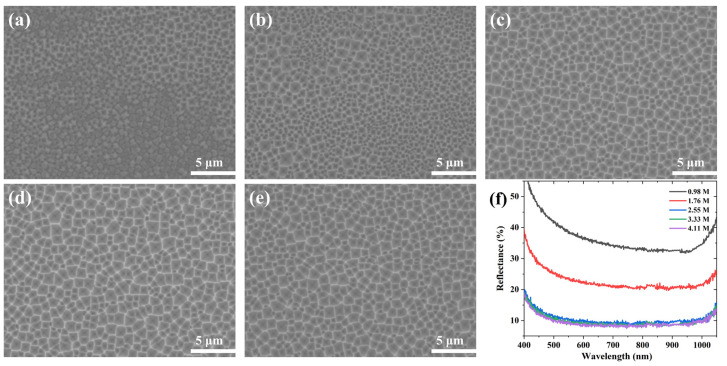
SEM images of Si surface etched in different H_2_O_2_ solutions for 7 min: (**a**) 0.98 M; (**b**) 1.76 M; (**c**) 2.55 M; (**d**) 3.33 M; (**e**) 4.11 M. (**f**) Reflectance spectra.

Figure 3 shows the SEM images and reflectance spectra of the samples etched with different concentrations of H_2_O_2_ solution at 0.5 M NH_4_HF_2_ for 7 min. When the concentration of H_2_O_2_ was 0.98 M, the wafer surface was filled with small pits and only a small area of the wafer surface had an evident inverted pyramid structure, as shown in Figure 3a. The overall structure was slightly improved as the H_2_O_2_ concentration was raised (Figure 3b). After the H_2_O_2_ concentration increased to 2.55 M, the silicon inverted pyramids were relatively uniformly distributed, the depth increased, and the four edges became clearly visible (Figure 3c–e). The reflectance spectra corresponded to the SEM images (Figure 2f), and the wafer etched with a low concentration of H_2_O_2_ (0.98–1.76 M) had a poor structure and higher reflectance, while the wafer etched with a high concentration of H_2_O_2_ (2.55–4.11 M) had a uniform structure and lower reflectance. The changes in reflectivity were less significant when the H_2_O_2_ concentration was 2.55–4.11 M.

When the concentration of H_2_O_2_ as an oxidizer was too low, it failed to provide enough holes to oxidize the surface, resulting in a significant decrease in the etching uniformity and etching rate of NH_4_HF_2_ and a large number of small inverted pyramids. PVP adsorbed on the silicon surface, resulting in a certain effective concentration of H_2_O_2_ touching the silicon surface, which inhibited the oxidation reaction. Therefore, increasing the concentration of H_2_O_2_ did not have a notable effect on the inverted pyramid structure. In conclusion, 2.55 M of H_2_O_2_ was sufficient for the experiment.

**Figure 4:**
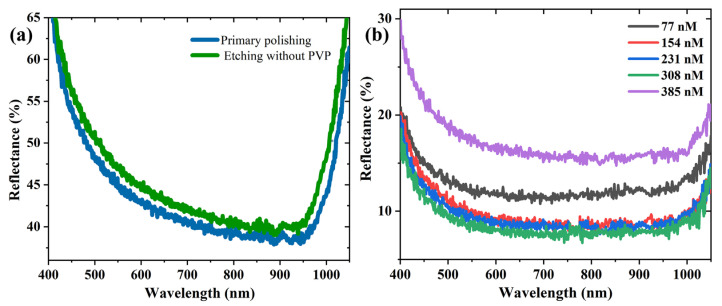
Reflectance spectra of etching systems etched for 7 min (**a**) without PVP and (**b**) with different concentrations of PVP.

Furthermore, PVP was necessary to form the inverted pyramid structure. The surface of the wafer became brighter and flatter upon immersing it in the PVP-free etch solution for 7 min. As seen in Figure 4a, the average reflectivity of the etched wafer reached 48.72%, which was higher than that of the primary polished wafer (46%). Figure 4b shows the reflectance spectra of the silicon etched with different concentrations of PVP. As shown in Figure 4b, the reflectance spectra showed an ascent after an initial decline trend with the increase in the PVP concentration. The average reflectivity was low when the PVP concentration was in the range of 154–308 nM, and the optimal anti-reflection performance of the wafer was achieved at a concentration of 308 nM. Considering this balance, we selected the PVP of 308 nM for the subsequent study.

**Figure 5:**
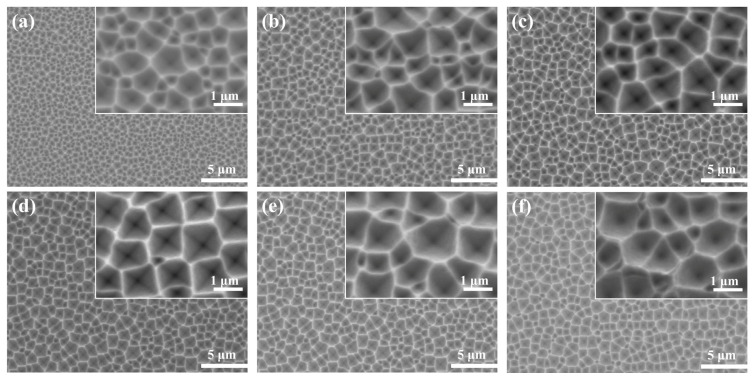
SEM images of Si surface etched in different AgNO_3_ solutions for 7 min: (**a**) 0.14 mM; (**b**) 0.24 mM; (**c**) 0.33 mM; (**d**) 0.42 mM; (**e**) 0.52 mM; (**f**) 0.62 mM.

Figure 5 shows the SEM images etched at different AgNO_3_ concentrations for 7 min. When the concentration of AgNO_3_ was low (0.14 mM), the silicon surface was covered with small-sized inverted pyramids, as shown in Figure 5a. The size of the inverted pyramid structure was enlarged, the depth expanded, and the edges became visible when increasing the AgNO_3_ concentration. The overall structure of the inverted pyramid did not change dramatically when the AgNO_3_ content was at 0.24–0.42 mM, but the uniformity improved and the proportion of inverted pyramids below 1 μm decreased substantially (Figure 5b–e). Further increasing the AgNO_3_ concentration did not promote the formation of the inverted pyramid. It was easy to identify that the depth of the inverted pyramid became shallow and it also tended to form shallow craters (Figure 5f).

**Figure 6:**
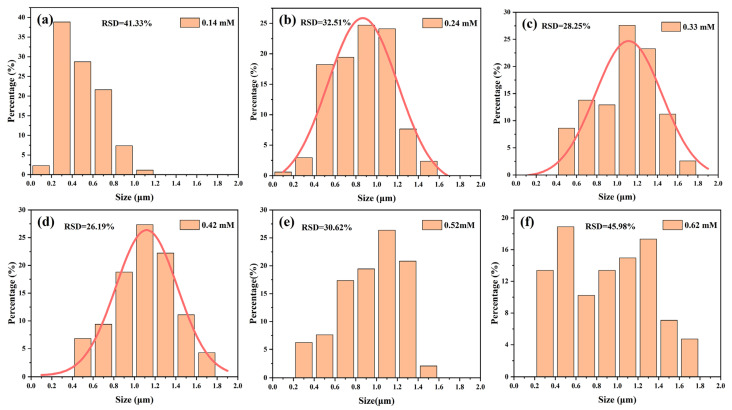
Histograms of inverted pyramid pore size distribution etched with different AgNO_3_ concentrations: (**a**) 0.14 mM; (**b**) 0.24 mM; (**c**) 0.33 mM; (**d**) 0.42 mM; (**e**) 0.52 mM; (**f**) 0.62 mM.

While the AgNO_3_ concentration was at 0.24–0.42 mM, the average reflectivity of the wafers was extremely similar (Figure S2). To better visualize the changes in the silicon surface morphology, ImageJ was applied to obtain metric statistics on the size and number of inverted pyramids, as illustrated in Figure 6. According to Figure 6a,f, it was apparently observed that the number of small-sized pyramids was 98% and 55% for low and high concentrations of AgNO_3_, respectively. Figure 6b–e show that the size distribution of the inverted pyramids was relatively even, especially in Figure 6d, where the size distribution was most concentrated, with a minimum relative standard deviation (RSD) of 26.19% and consistent with the Gaussian distribution. Therefore, 0.42 mM AgNO_3_ was selected for further experiments.

**Figure 7:**
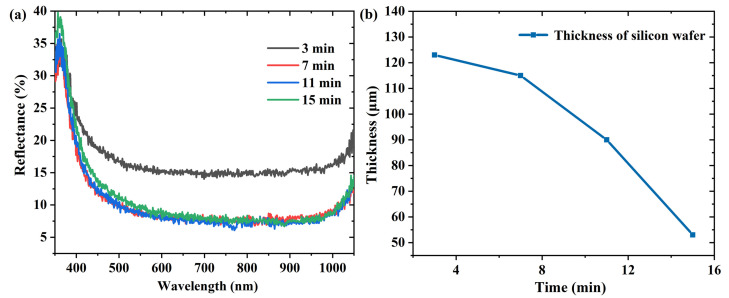
(**a**) Reflectance spectra and (**b**) thickness variation of silicon wafers etched for different times.

Figure S3 shows the SEM images of the silicon surface etched at different times. As illustrated in Figure S3a, the inverted pyramid structure fabricated at 3 min was evidently shallow, the four side edges of the inverted pyramid were blurred, and the structure size uniformity was poor. As the etching time increased, the size of the inverted pyramids became significantly larger (Figure S3b–d). On increasing the etching time, the average reflectivity descended to a stable level (Figure 7a). This indicates that a longer etching time does not further improve the anti-reflective properties of the wafer. In addition, the weight loss of the wafer was too severe for a long etch time, and the thickness of the wafer was less than one third of the original thickness after 15 min. The wafer became more brittle, which was not conducive to subsequent operations. Hence, we set a total of seven minutes as the most appropriate etching time.

### 3.2. SERS Response from the Textured Surface

An aqueous solution of 0.5 M NH_4_HF_2_, 2.55 M H_2_O_2_, 308 nM PVP, and 0.42 mM AgNO_3_ was chosen for the experiment considering potential advantages in size uniformity and anti-reflection properties. The etching temperature was set to 65 °C and the total etching time was 7 min. The silicon inverted pyramids prepared with these parameters were subjected to the subsequent deposition of silver nanoparticles.

Regarding the SERS response, we focused on comparing the Raman peak intensities of silver nanoparticles on silicon surfaces at different deposition states using two different methods of depositing silver nanoparticles.

**Figure 8:**
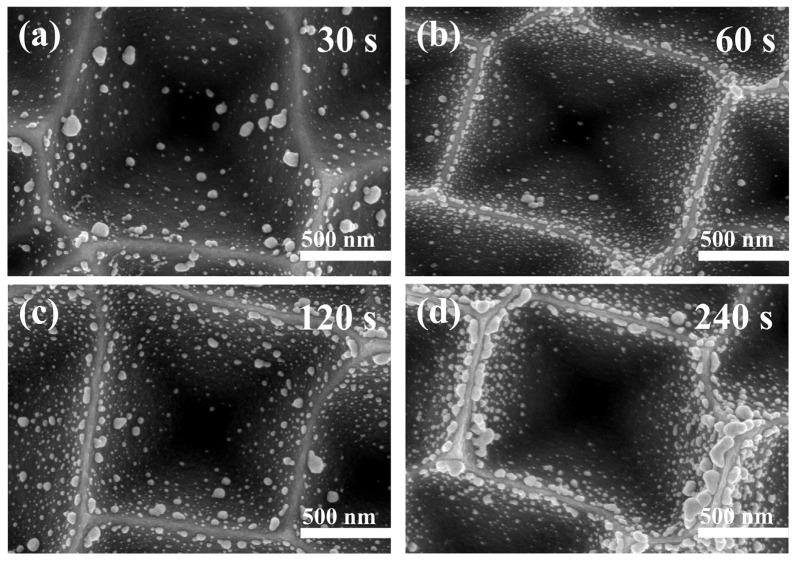
SEM images of substrates immersed in AgNO_3_/HF (0.5 mM AgNO_3_, 9% HF) aqueous solution for different times at room temperature: (**a**) 30 s; (**b**) 60 s; (**c**) 120 s; (**d**) 240 s.

Figure 8 shows the SEM images of silver nanoparticles deposited at different times by electroless deposition. We identified that the silver nanoparticles were not easily deposited to the bottom of the inverted pyramid and the particle size gradually became smaller along the silicon wall. As the deposition time increased, the number of silver nanoparticles on the silicon surface was boosted, and the silver nanoparticles tended to undergo aggregation at the top of the silicon wall, transforming into nano-chain-like silver. Figure S4 shows the EDS spectrum of the substrate deposited with 240 s of Ag NP_S_, confirming the presence of only three elements, C, Si, and Ag, which proves that no other elements were present in the Ag nanoparticles.

**Figure 9:**
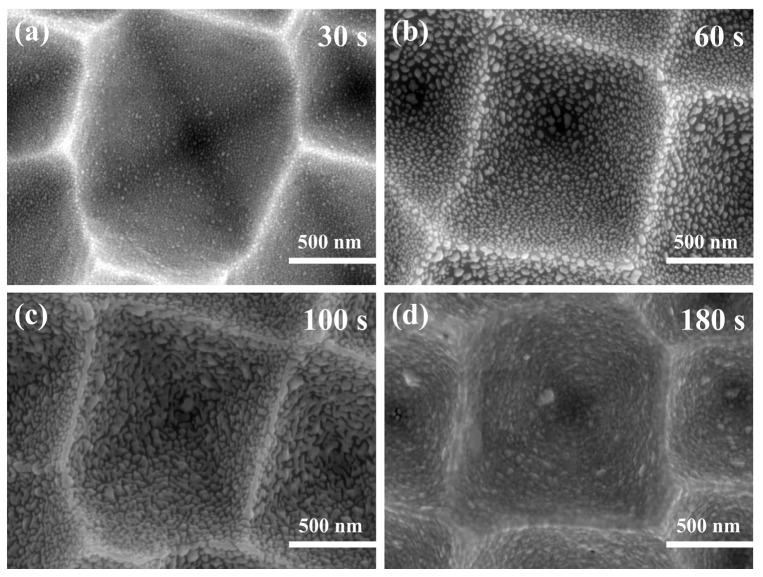
SEM images with magnetron sputtering at different times: (**a**) 30 s; (**b**) 60 s; (**c**) 100 s; (**d**) 180 s.

Figure 9 shows SEM images of silver nanoparticles sputtered on silicon inverted pyramids with a radiofrequency sputtering system at different times. When the sputtering time was 30 s, the silver nanoparticles were deposited regularly on the silicon inverted pyramids, without aggregation or excessive spacing. The size and spacing of the silver nanoparticles were 8–20 nm and 5–10 nm, respectively (Figure 9a). After extending the sputtering time to 60 s, the size of the silver nanoparticles ranged from 10 to 45 nm, and the majority of them were above 20 nm. Meanwhile, it was evident that the distance between the particles became more significant (Figure 9b). Upon continuing to increase the time, the silver nanoparticles gradually transformed into networked silver (Figure 9c) and became a silver film at a sputtering time of 180 s (Figure 9d). Figure S5 illustrates the EDS spectrum of the substrate sputtered with Ag NPs for 30 s, showing the presence of only three elements, O, Si, and Ag, which proved that no other elements were present in the Ag nanoparticles. Silver nanoparticles and silicon are easily oxidized in air, resulting in the presence of the O element.

**Figure 10:**
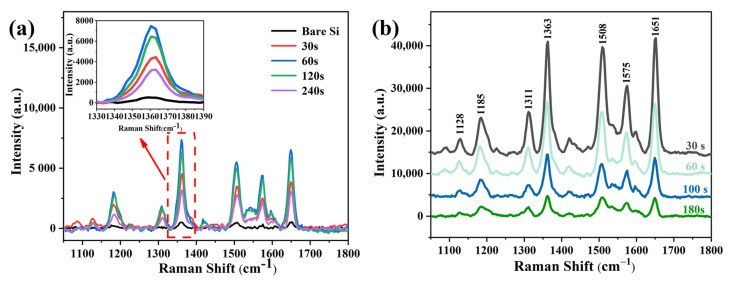
Raman spectra of R_6_G measured on SERS substrates prepared with (**a**) different Ag deposition times and (**b**) different Ag sputtering times.

Figure 10a shows the Raman spectra of 0.1 M R_6_G tested on bare silicon and 1.0 × 10^−6^ M R_6_G measured on silicon inverted pyramid SERS substrates after depositing Ag NPs by electroless deposition for different times. The Raman peak positions of R_6_G have been labeled at 1128, 1185, 1311, 1363, 1508, 1575, and 1651 cm^−1^, which are consistent with the previously reported peak positions [34]. The 1128, 1182 cm^−1^ SERS peaks are attributed to C-H in-plane bending. The SERS peaks at 1311 cm^−1^ can be attributed to N-N in-plane bending vibration, while the SERS peaks at 1363, 1508, 1575, and 1651 cm^−1^ are all due to aromatic C-C stretching vibration [35]. It can be clearly seen that all the SERS substrates show high-intensity Raman peaks compared to bare silicon, which proves the feasibility of silicon inverted pyramid/Ag NPs as SERS substrates. In addition, considering that the most dominant peak was around 1363 cm^−1^, this peak was chosen to compare the SERS performance of different substrates.

As the depositing time increased from 30 to 60 s, the intensity also surged. This was due to the increase in the number of silver nanoparticles deposited on the silicon walls, which reduced the gap between the particles, strengthening the electric-field enhancement and the associated hot spots. After increasing the deposition time to 120 s, the intensity of the peaks only slightly decreased, which was consistent with the similar results in the SEM images. Nevertheless, at the deposition time of 240 s, a significant decrease in the intensity of the peak at 1363 cm^−1^ could be observed. Due to the aggregation of isolated nanoparticles into silver nanostrands with increasing time, the size and spacing of the silver nanoparticles increased considerably, making it difficult to generate strong electric-field coupling and reducing the Raman peak intensity. Thus, a deposition time of 60 s was set as the ideal parameter for optimal substrate SERS performance.

Figure 10b shows the SERS spectra of 10^−6^ M R_6_G molecules of the substrate after sputtering silver nanoparticles at different times. As illustrated, the intensity of the peak gradually becomes weaker as the sputtering time increases. This is due to the longer sputtering time, which hampers the formation of silver nanoparticles with a smaller size and spacing at the silicon wall, weakening the electric-field enhancement and the associated hot spots. Nevertheless, the substrates prepared by the radiofrequency sputtering system show superior SERS performance compared to those prepared by electroless deposition. Furthermore, the intensity of the peak at 1363 cm^−1^ of the substrates sputtered for 30 s was considerably higher than that of the substrates prepared by the optimal electroless deposition (60 s). This is because the radiofrequency sputtering system can build smaller and denser silver nanoparticles compared with electroless deposition, solving the issue of electroless deposition of silver nanoparticles that cannot be easily deposited at the bottom of the inverted pyramidal silicon wall and tend to accumulate at the top.

**Figure 11:**
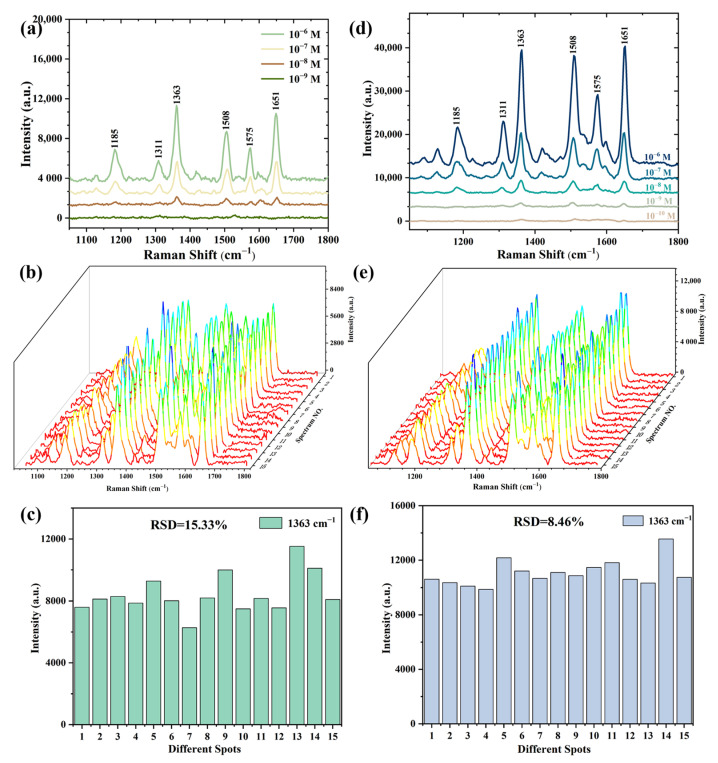
(**a**) The Raman spectra of various concentrations of R_6_G solution; (**b**) Raman spectra of 10^−6^ M R_6_G solution from 15 random points; (**c**) intensity of the characteristic peak at 1363 cm^−1^ from 15 random points on SERS substrates prepared by electroless deposition for 60 s; (**d**) the Raman spectra of various concentrations of R_6_G solution; (**e**) Raman spectra of 10^−7^ M R_6_G solution from 15 random points; (**f**) intensity of the characteristic peak at 1363 cm^−1^ from 15 random points on SERS substrates prepared by sputtering for 30 s.

High sensitivity and reproducibility are crucial for an excellent SERS substrate. Therefore, the SERS substrates prepared by different methods were tested for their limit concentration and reproducibility, respectively, and the results are shown in Figure 11. Figure 11a shows the Raman spectra of various R_6_G concentrations (10^−6^~10^−9^ M) collected from the substrates prepared by electroless deposition; the lowest detectable concentration reaches 10^−8^ M. To evaluate the reproducibility for the SERS signals, Raman spectra of the 10^−6^ M R_6_G were collected from 15 different points on the substrates (Figure 11b). Figure 11c shows the peak intensities at 1363 cm^−1^ for R_6_G with a concentration of 10^−6^ M from 15 different points on the SERS substrate prepared by electroless deposition, and the relative standard deviation (RSD) was calculated to be 15.33%. Sensitivity and repeatability tests and calculations were also conducted for the substrates prepared by radiofrequency sputtering, and the results are shown in Figure 11d,e. This result demonstrates that the detection limit of a substrate prepared by sputtering for 30 s for the R_6_G solution can reach 10^−10^ M. The Raman signal of 10^−7^ M R_6_G on the substrate was collected and the results indicate higher reproducibility with an RSD of 8.46%.

To intuitively evaluate the SERS effects of different substrates, the enhancement factor (EF) can be expressed by calculating the following Equation (1):

(1) $$EF=\frac{I_{SERS}\times N_{RS}}{I_{RS}\times N_{SERS}}$$

where I_SERS_ and I_RS_ are the peak intensities at 1363 cm^−1^ for R_6_G obtained from SERS spectra and normal Raman spectra, respectively. N_SERS_ and N_RS_ are the numbers of R_6_G molecules excited by the laser beam on the SERS substrate and bare silicon wafer, respectively. Therefore, the EF values of the SERS substrates prepared by electroless deposition and radiofrequency sputtering were 1.68 × 10^6^ and 2.21 × 10^7^, respectively. The value for the SERS substrate fabricated by radiofrequency sputtering is 13.07 times higher than that of electroless deposition.

**Figure 12:**
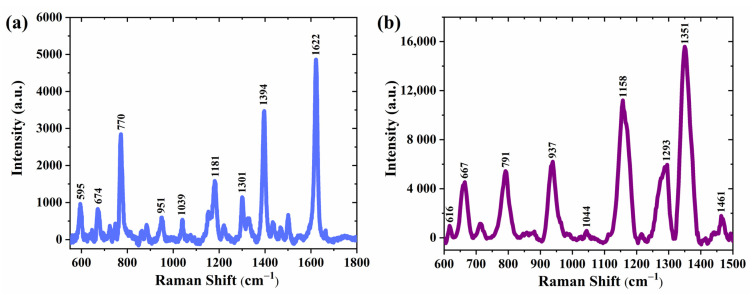
Raman spectra of (**a**) MB (1.0 × 10^−5^ M) and (**b**) AMX (1.0 × 10^−4^ M) were collected on a substrate (silver sputtering time of 30 s on Si-inverted pyramid).

In addition, to verify the general sensitivity to other organic molecules, we also selected MB and AMX molecules to detect on the substrates, which were prepared by sputtering silver nanoparticles on Si-inverted pyramids. Figure 12a,b shows the Raman spectra of 10^−5^ M MB solution and 10^−4^ M AMX solution collected on the SERS substrate sputtered with 30 s silver nanoparticles, respectively. For comparison, the normal Raman spectra of 1 mM MB and 0.01 M AMX on bare silicon were also collected for each one (Figure S6a,b). All characteristic distinguishable peaks of MB positioned at 595, 674, 770, 951, 1039, 1181, 1301, 1394, and 1622 cm^−1^ and AMX positioned at 616, 667, 791, 937, 1158, 1293, 1351, and 1461 cm^−1^ were observed, which are in good agreement with previously reported values, and the characteristic peaks are greatly enhanced compared to normal Raman spectra. The corresponding vibration mode attributed to each Raman peak for both analytes is provided in Tables S1 and S2. In this study, only the effect of different silver nanoparticle deposition states on SERS was investigated, leaving room for further examinations focused on the effect of different sizes of inverted pyramids on the performance of SERS substrates.

## 4. Conclusions

We prepared silicon inverted pyramids with excellent anti-reflectivity and uniform sizes by Ag@PVP-ACE. The significant Raman enhancement of R_6_G by silicon inverted pyramid/Ag NPs as SERS substrates was demonstrated. Comparing the Raman enhancement effects of SERS substrates in different silver nanoparticle deposition states, our most important outcomes showed that silver nanoparticles that are smaller and more densely distributed lead to the formation of substrates with higher sensitivity. The silicon inverted pyramid/Ag NPs prepared in this study exhibit a similar size and structure with a denser distribution of inverted pyramids compared to commercial Klarite SERS samples, as well as other advantageous features, e.g., lower costs, which are extremely beneficial for practical applications of SERS substrates.

## Data Availability

Not applicable.

## Supplementary Materials

The following supporting information can be downloaded at: https://www.mdpi.com/article/10.3390/ma16103634/s1.
