# Supplementary material for: A Simple Method for the Fabrication of Silicon Inverted Pyramid Substrates for Surface-Enhanced Raman Spectroscopy
Source: Materials (Basel). 2023 May 10;16(10):3634. doi: 10.3390/ma16103634 (PMC10220623; doi:10.3390/ma16103634)
Supplement: Supplementary file 1 [file materials-16-03634-s001.zip › materials-2384338-supplementary.pdf]

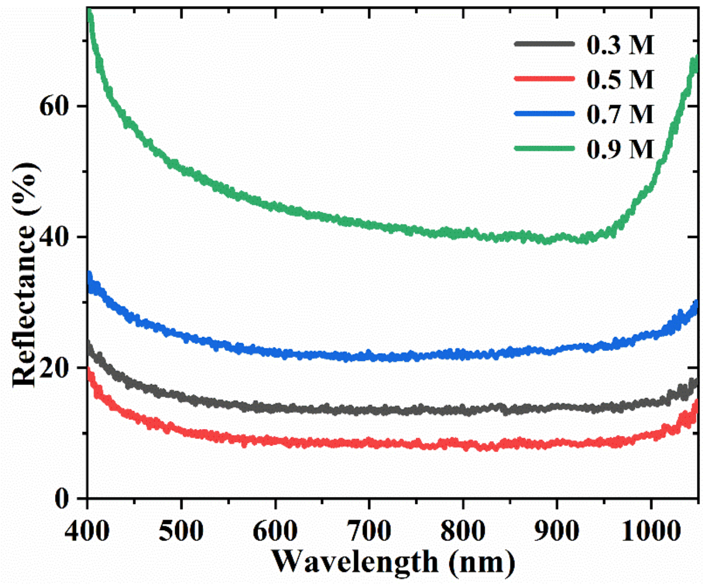

Figure S1. Reflectance spectra of silicon wafers etched in different NH_4HF_2 solution for 7 min.

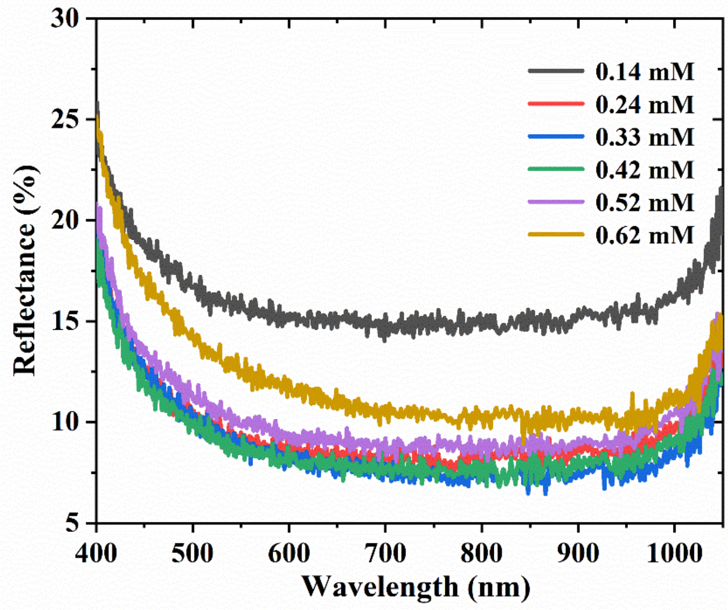

Figure S2. Reflectance spectra of silicon wafers etched in different $AgNO_3$ solution for 7 min.

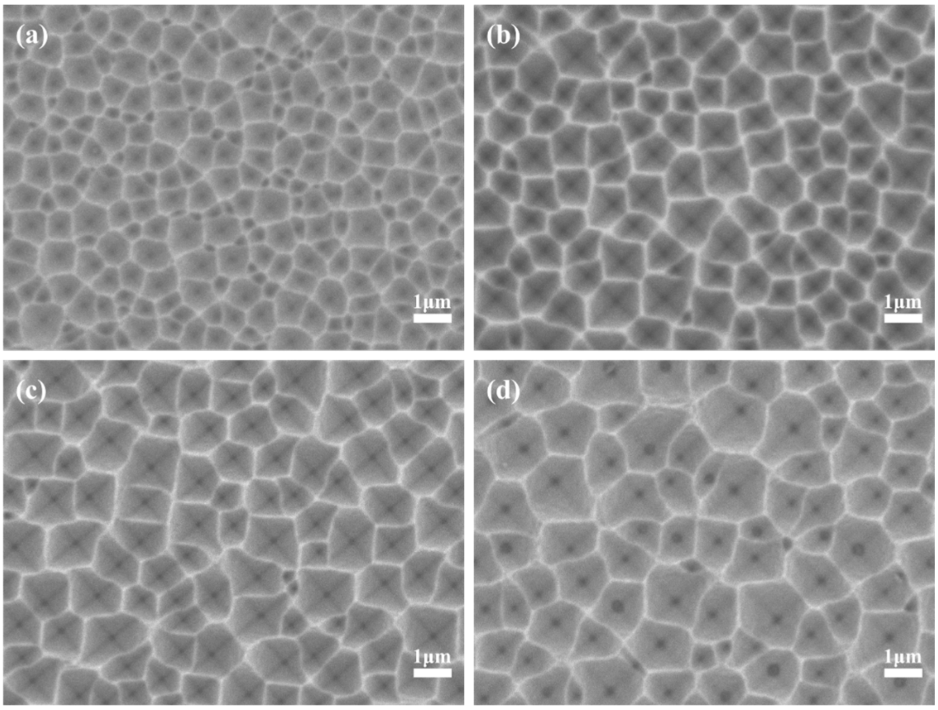

Figure S3. SEM images of silicon surface etched for different times (a) 3min; (b) 7min; (c) 11min; (d) 15min.

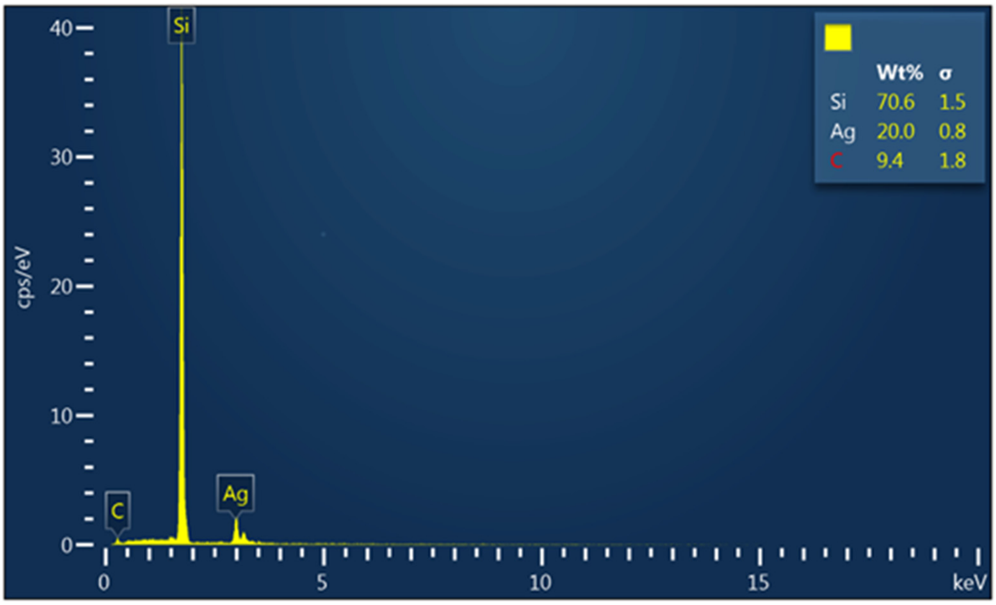

Figure S4. EDS spectrum of the substrate deposited with 240 s of Ag NPs

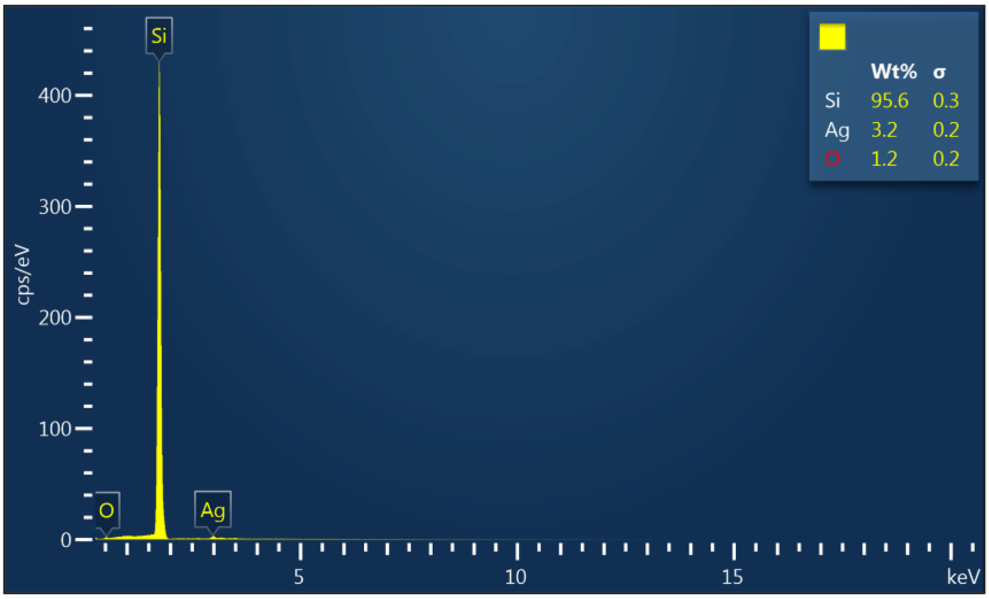

Figure S5. EDS spectrum of the substrate sputtered with 30 s of Ag NPs

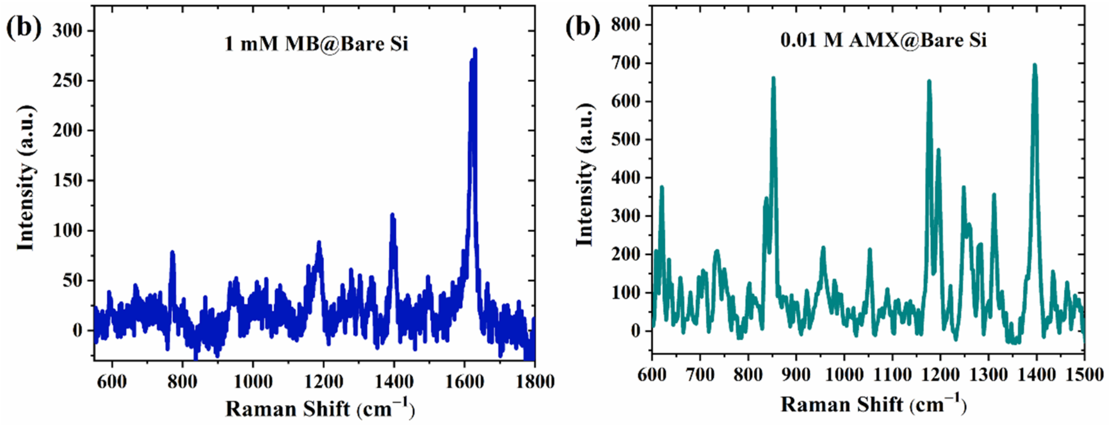

Figure S6. Real time Raman Spectra of MB (1 mM) and MP (0.01 M) were collected from Bare Si

Table S1. Assignment of vibration modes corresponding to each Raman peak for probe molecules methylene blue

Raman peak (cm^{-1})	Band assignment
595	Skeletal deformation of C-S-C
674	Out-of-plane bending of C-H
770, 951, 1039	In-plane bending of C-H
1181,	Stretching of C-N
1301	In-plane ring deformation of C-H
1394	Symmetrical stretching of C-N
1622	Ring stretching of C-C

Table S2. Assignment of vibration modes corresponding to each Raman peak for probe molecules amoxicillin

Raman peak (cm^{-1})	Band assignment
616	Bending of -OH
667	Ring deformation of benzene
791	In-plane deformation
937	Amine bending
1158	Benzene ring’s C−H bending
1293	Benzene’s in-plane deformation
1351	Amine’s twisting
1461	Asymmetric bending of CH_3
